# Sophisticated self‐circumcision: Case report

**DOI:** 10.1002/ccr3.3282

**Published:** 2020-10-06

**Authors:** Vincent Nagy

**Affiliations:** ^1^ Department of Urology P.J. Šafárik University Medical Faculty and L. Pasteur University Hospital Košice Slovak Republic

**Keywords:** bleeding, complications, psychological problem, redundant foreskin, self‐circumcision

## Abstract

In this case report, we describe a unique case of a highly motivated patient who tried to solve his problem with a redundant foreskin by a sophisticated, thoughtful method. Dissatisfaction with the foreskin made it difficult for him to establish intimate contact. After studying the operation on the Internet, he performed self‐circumcision.

## Graphical abstract

The appearance of the penis after a failed self‐circumcision.

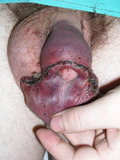

## 1 INTRODUCTION

In this case report, we describe a unique case of a highly motivated patient who tried to solve his problem with a redundant foreskin by a sophisticated, thoughtful method. Dissatisfaction with the foreskin made it difficult for him to establish intimate contact. After studying the operation on the Internet, he performed self‐circumcision.

Circumcision is one of the most common surgical operations performed worldwide for traditional, ritual, religious, or health reasons. In our cultural environment, in Slovakia in Central Europe, circumcision is most often performed in patients with phimosis in hospital facilities. Phimosis makes it difficult to maintain adequate hygiene and can lead to infections as well as penile cancer under the appropriate conditions.^1^ There are other foreskin pathologies, including cosmetic reasons that may cause problems associated with sexual matters. These situations are generally the main motivation for adult patients to undergo surgical circumcision.^2^ In this case report, we describe a unique case of a highly motivated patient who tried to solve his redundant foreskin problem by self‐circumcision using a sophisticated, thoughtful method. Concerns about surgery and feelings of shame or humiliation from being in the hospital were the main reasons why the patient performed the operation by himself.

## 2 CASE REPORT

**FIGURE 1:**
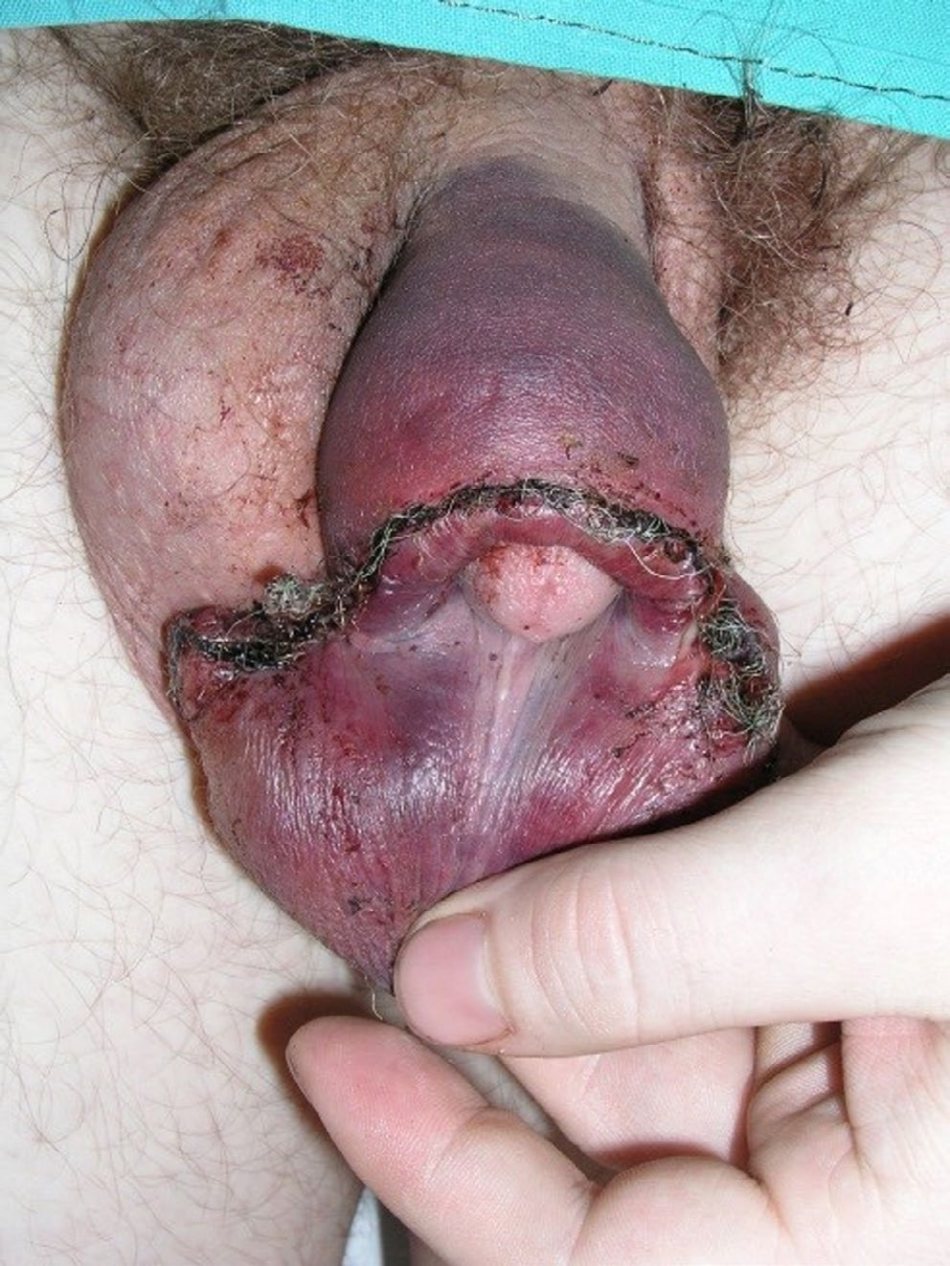
The photograph documents the local finding of the surgical wound on the second day after arrival at the hospital

The patient, a 25‐year‐old electrical engineering student, was above average mentally and physically advanced. Psychiatric examination did not reveal any signs of a mental disorder with signs of self‐harm. His technical education had a decisive influence on the choice of sewing material. His actions were based on rational consideration due to dissatisfaction with the look of his redundant foreskin. There was an excess of foreskin during both flaccid and erect states, which bothered him during sexual intercourse. Dissatisfaction with the foreskin made it difficult for him to establish intimate contact, so he decided to solve his problem. However, due to feelings of shame, he did not seek professional help, but instead decided to perform the foreskin surgery by himself. Using various websites from the Internet, he found a simplified procedure of an operation with illustrative examples utilizing rubber equipment to cause vasoconstriction. He considered self‐circumcision only a technical problem. The procedure was planned to be undertaken in about a month, and he used this time to prepare for the operation. Since he lived in a student house with common areas for all residents, he performed the operation at the end of the week when the other residents had returned home. Based on this information, he acquired the necessary tools. He was aware of the necessity to solve the problems of sterility, pain, and bleeding during surgery. For suture material, he used a fine wire made of stainless steel with a galvanized surface. He bought sterile rubber gloves and an alcohol‐free iodine solution (Betadine) from a pharmacy. He made a vasoconstrictor rubber ring for his penis in order to minimize foreskin bleeding. The suture materials, fine stainless galvanized wires, were steamed in boiling water to achieve sterility. He then put the wires through a regular straight needle. He made the incision with a razor blade, which he also sterilized by boiling. To prevent major bleeding, he immediately sutured the foreskin after a short incision. Pain was reduced by the use of an ethylene oxide spray, which is used in sports medicine. He continued like this along the dorsal circumference of the foreskin. Thus, he proceeded on the dorsal half of the foreskin (Figure 1). During the procedure, the vasoconstrictive rubber ring came loose and the patient began to bleed. Also, the operation in his administration took longer than expected. He was frightened, and the self‐circumcision was interrupted. The operation began in the afternoon around 4 pm Due to continued bleeding and pain, he stopped partial self‐circumcision and visited the hospital in the evening on the same day. He travelled to hospital via public transport using a tram. The patient alleviated the bleeding from the wound by applying pressure with a towel. His genitals were wrapped in a towel. The wound was bleeding weakly. He had swelling and hematomas around the incision, which spread from the penis to the scrotum. There were no signs of infection in the surgical wound. After admission to the urology department of the Medical Faculty of PJ Šafárik University in Košice, the patient received prophylactic antibiotics, and the day after the standard examination, circumcision was performed under regional anesthesia.

## 3 DISCUSSION

Individual case reports of patients who have attempted self‐circumcision for various reasons are described in literature. It was not performed with self‐mutilating intent due to a mental disorder. But rather it was a thoughtful activity based on a subjective evaluation of perceived changes in genitalia. In some countries, they are performed for traditional reasons. Self‐circumcision without adequate hygienic conditions using various sharp instruments is more common in some traditional areas of Africa where circumcision is a ritual ceremony.^3^ Other younger patients have self‐circumcised with a razor blade for subjective excess foreskin, or for other reasons. In these cases, surgeries were performed without using any anesthesia. Unpleasant complications may include massive hemorrhage, possibly with laceration or penile skin avulsion.^4^, ^5^ Some European or US patients have misused either uncertified or other recommended and commercially available devices from online stores. These devices when used, after the two rings are properly applied inside and outside of the foreskin, halt blood flow to the foreskin by the pressure formed. After a few days of bloodless necrosis, the foreskin can be removed by a sharp object, such as a scalpel or scissors.^6^, ^7^

The most common complications, especially in the use of sharp tools (razor blade and the like), in addition to the bleeding and laceration of the penis already mentioned, were urethral injury, infection, penile curvature, stinging, or necrosis of the skin and penile tissues.^6^

Our patient differed from other patient case reports in the literature, due to his sophisticated approach to self‐circumcision. He was aware of the dangers of the operation, which consisted in the use of instrumentation, suture material, sterility, and asepsis in his own performance, as well as problems with perception of pain or bleeding. Although the solution to foreskin pathology or dissatisfaction is readily available in any healthcare facility, the patient opted to operate by himself. Finally, he understood the health risks, and immediately after the occurrence of unsolvable complications, he sought professional help from the hospital.

## 4 CONCLUSION

In this case report, we describe a unique case of a highly motivated patient who tried to solve his problem with a redundant foreskin by a sophisticated, thoughtful method. Dissatisfaction with the foreskin made it difficult for him to establish intimate contact. After studying the operation on the Internet, he decided to perform self‐circumcision. During surgery, he used sterile equipment including: straight needles; nonalcoholic disinfectant solution containing iodine; local anesthesia by ethylene oxide; and fine, galvanized, stainless steel wires from a conductor. After unresolved complications and bleeding, he sought professional help from the hospital. There were no signs of infection in the surgical wound. This case report points to patients in the practice of a general practitioner or urologist who, if they are not satisfied with their genitals and their problem is not satisfactorily solved, can solve their problem in an extreme way—by self‐circumcision.

Attempts at self‐circumcision for any reason are very rare in our country. Nevertheless, there are occasional individuals who, because shameful feelings, prefer a distinct solution. After experiencing the reality of the procedure in these unsuccessful attempts, and especially the complications, the patients are forced to seek professional help.

## CONFLICT OF INTEREST

No conflict of interest was declared by the author.

## AUTHOR CONTRIBUTIONS

VN: took the lead in patient management, did the photography, and wrote the drafts and final article.

## ETHICAL APPROVAL

Written consent was obtained from the patient to take photographs and to publish.
